# Supplementary material for: Temporal and spatial dynamics of Plasmodium falciparum clonal lineages in Guyana
Source: PLoS Pathog. 2024 Jun 13;20(6):e1012013. doi: 10.1371/journal.ppat.1012013 (PMC11206942; doi:10.1371/journal.ppat.1012013)

Supplementary - Temporal and spatial dynamics of *Plasmodium falciparum* clonal lineages in Guyana

#

# **Materials and methods**

## **Sample collection and mapping of epidemiological zone**

For 2016-2017, samples were collected as part of the malaria routine surveillance system implemented in Guyana [30]. Health care facilities were in charge of collecting anonymized *P. falciparum* positive cases. Identification of individuals cannot be established. In accordance with WHO guidelines on ethical issues in public health surveillance the sample collections were exempt of Ethics Review Committee since these interventions were part of the Malaria Control Program defined by the Ministry of Public Health of the country as monitoring of public health programs (<https://www.who.int/ethics/publications/public-health-surveillance/en>). Written informed consent was obtained from all study participants or their parents or guardians. The analysis of the samples was also approved by the Environmental Protection Agency in the frame of the Nagoya Protocol on Access to Genetic Resources and the Fair and Equitable Sharing of Benefits Arising from their Utilization. Regarding 2018-2021 samples, this study was approved by a local ethical committee in Guyana (Institutional Review Board, no. 645/2019) as well as the Harvard Longwood IRB (IRB19-1779).

# **Results**

### **Spatial Clonal Dynamics in Guyana**

Most highly related clusters were widely distributed and not restricted to a particular geographic location (Fig C in S1 Text). This suggests that these clones can circulate rapidly across the country. However, they seem to be preferably associated with a particular epidemiological zone. Lower Mazaruni appeared as the location where most highly related clusters were circulating. The majority of patients reported travel to this epidemiological zone. In 2016-17, seven highly related clusters were circulating (Cluster 1, 2, 4, 7, 8, 10, 11). For instance, samples from Cluster 1 were principally recovered over the 2016-17 study period (83.0%, n = 93 samples). This cluster was composed mainly of a large single clone (C#1, Fig 3) and was predominantly observed in Lower Mazarudi (40.7%) and Lower Cuyuni (20.1%). On the other hand, Cluster 6 (n = 63) was predominantly found in Potaro (n=41, 65.1%) and Upper Cuyuni (n=10, 15.9%) (Fig C in S1 Text). Three highly related clusters were more prevalent in 2020-21 (cluster 3, 4 and 11). Cluster 3 and cluster 4 were present at low frequency in 2016-17, 17.6% (n_2016-17_=27 samples) and 6.8% (n_2016-17_=5 samples) respectively (Table 2). The clones in cluster 3 were observed in six epidemiological zones in 2016 indicating that these closely related clones have been circulating in Guyana for some time (Fig C in S1 Text) with samples found in Lower Mazaruni (n=30, 23.4%), Potaro (n=30, 23.4%) and Lower Cuyuni (n=25, 19.5%). This cluster included two large related C#100 (n=28) and #61 (n=16) which persisted throughout the study (average IBD of 0.591 – Fig 3). Cluster 4 was found in nine epidemiological zones and was mainly associated with lower and mid Mazaruni (56.5% and 12.9% respectively) while cluster 9 was found principally in Lower Mazaruni (45.6%, n=17). Finally, two highly related clusters were found in similar proportion across the two study periods (Cluster 6 and 13). Cluster 6 was found in 14 epidemiological zones. However, in 2016-17, most of the samples were in found in Essequibo (50.0% of samples in 2016-17, n=12), while in 2020-21, samples were predominantly found in Potaro (31.4% of samples in 2020-21, n=11), Lower Cuyuni (17.1%, n=6) and Kaituma and Barima (17.1%, n=6). On the other hand, Cluster 13 was mainly associated with Lower Mazaruni (n=25, 41.0%) and Lower Cuyuni (n=12, 19.8%) in similar proportions across the two study periods.

**Detailing selection signals from isoRelate**

Among short-lived clones (duration under three months), the selection signal was fainter but similar to the spectrum in long-lasting clones except for the peak on chromosome 9. We found among a total of 13 segments encapsulating 283 genes. In 2020-21, signals of positive selection did not appear as strong as in 2016-17. However, 19 segments were found (425 genes) for clones lasting less than three months. In the 49 clones which were sampled over more than three months, a positive signal of selection was only found in 11 segments (289 genes). No strong signal was observed in long-lasting clones compared to short-lived ones except on a segment on chromosome 5.

When comparing selection signals among long-lasting clones over the two periods 2016-17 and 2020-21, a signal on chromosome 7 that included the *pfcrt* (PF3D7_0709000) was conserved (Fig 6, J in S1 Text). When investigating mean pairwise IBD patterns using an overlapping window across chromosome 9 (Fig I in S1 Text), the presence of high IBD segments were observed over the first peak of this chromosome (chr9: 61,342-208,725) in samples from 2020-2021. For the eleven NSY which increased in the genomic region, MAF values were around 0.29 (± 0.05). Having these mutations resulted in an increase in clonal duration (Fig 5E-F). Under this MAF window, the average clonal duration was 98 days and clones having these mutations lasted between 111 and 130 days. However, these polymorphisms did not have an impact on the abundance of clones. The *pfkic6* Q1680K mutation on chromosome 6 belonged to this MAF window (MAF = 0.288) and led to an average clonal duration of 115 days.

The selection signal on chromosome 2 and at the end of chromosome 4 in 2016-2017 was not found five years later (Fig J in S1 Text). In 2020-2021, selection signals appeared in the middle of chromosome 4 which included PF3D7_0415800 (*pfpic3* - chr4:693,250-696,792), a PhIL1 interacting candidate which appeared to be a genomic region relatively conserved across the dataset (Fig J in S1 Text). Other genomic regions included a segment on chromosome 5 and 6 which harboured PF3D7_0629500 (AAT1) as well as segments on chromosome 10, 12, and 14. In these segments, two genes on chromosome 10 increased in frequency XL2 (PF3D7_1001600 - exported lipase 2) and PF3D7_1004400, encoding for a RNA-binding protein or PSOP17 (PF3D7_1218800), a secreted ookinete protein on chromosome 11. To confirm the robustness of these analyses, another analysis was performed comparing singletons and a sample from each clone. Results obtained (Fig H in in S1 Text) were similar to the previously described analysis comparing long-lasting clones (sampled over three months – Fig. 7).

When comparing genome-wide selection signals of clones with *pfcrt* C350R (n=63) and clones harboring the wildtype (n = 96) stratified by the two time periods, the genomic region on chromosome 7 where *pfcrt* is located was under positive selection (Fig I in S1 Text). Clones with *pfcrt* C350R displayed signals of positive selection in 12 genomic regions (158 genes).

# Supplementary Tables

Table A – Emerging highly related clusters emerging in Guyana. Highly related clusters are defined as a group of at least 3 clones with an average IBD ≥ 0.40.

| cluster | **Mean IBD** | **Number of clones** | **Number of clones and singletons** | **Number of isolates** | **groups** |
| --- | --- | --- | --- | --- | --- |
| **1** | 0.798 | 7 | 21 | 107 | 1 |
|  | 0.082 | 0 | 2 | 2 | 2 |
| **2** | 0.544 | 15 | 46 | 148 | 3 |
| **3** | 0.548 | 18 | 43 | 147 | 4 |
| **4** | 0.696 | 5 | 11 | 70 | 5 |
| **5** | 0.763 | 3 | 10 | 64 | 6 |
| **6** | 0.654 | 5 | 5 | 62 | 7 |
| **7** | 0.434 | 22 | 83 | 194 | 8 |
|  | 0.292 | 0 | 5 | 5 | 9 |
| **8** | 0.51 | 8 | 18 | 71 | 10 |
| **9** | 0.53 | 4 | 17 | 44 | 11 |
| **10** | 0.481 | 6 | 11 | 35 | 12 |
|  | 0.215 | 2 | 12 | 13 | 13 |
| **11** | 0.485 | 5 | 17 | 42 | 14 |
|  | 0.569 | 2 | 6 | 32 | 15 |
|  | 0.334 | 15 | 50 | 86 | 16 |
|  | 0.37 | 11 | 30 | 64 | 17 |
| **12** | 0.401 | 8 | 18 | 61 | 18 |
|  | 0.327 | 15 | 44 | 69 | 19 |
| **13** | 0.409 | 10 | 44 | 93 | 20 |

Table B – Polymorphism which increased in frequency between 2016-2017 and 2020-2021

| **Gene** | **Chr:Position** | **Number of NSY in 99th percentile** | **Description** | **Codons** | **AA** |
| --- | --- | --- | --- | --- | --- |
| **PF3D7_0113800** | 1:527107-536351 | 3 | DBL containing protein, unknown function | 3436G>A; 3445C>T; 8458G>A | Glu1146Lys; His1149Tyr; Val2820Ile |
| **PF3D7_0216800** | 2:696193-699561 | 3 | TMEM121 domain-containing protein, putative | 1381T>A; 1373G>A; 938A>G | Cys461Ser; Ser458Asn; Gln313Arg |
| **PF3D7_0418600** | 4:834394-840429 | 3 | regulator of chromosome condensation, putative | 2254T>G; 2255G>T; 2270C>T | Gly748Asp; Cys752Gly; Cys752Phe |
| **PF3D7_0609000** | 6:370261-388494 | 1 | nucleoporin NUP637, putative | 12682G>C | Glu4228Gln |
| **PF3D7_0609700** | 6:413652-419781 | 1 | protein KIC6 | 5038C>A | Gln1680Lys |
| **PF3D7_0701900** | 7:79890-82943 | 4 | Plasmodium exported protein, unknown function | 2654T>A; 2091T>A ; 2075G>A ; 2013T>A | Ile885Lys; Asp697Glu; Ser692Asn; Asn671Lys |
| **PF3D7_0704000** | 7:167489-177295 | 1 | conserved Plasmodium membrane protein, unknown function | 2220A>C | Glu740Asp |
| **PF3D7_0723800** | 7:993232-1000233 | 1 | apicomplexan kinetochore protein 1, putative | 2129A>T | Glu710Val |
| **PF3D7_0828200** | 8:1216271-1220716 | 1 | leucine--tRNA ligase, putative | 3201A>T | Lys1067Asn |
| **PF3D7_0831600** | 8:1358314-1363618 | 1 | cytoadherence linked asexual protein 8 (CLAG8) | 4099A>G | Lys1367Glu |
| **PF3D7_0902400** | 9:106514-108406 | 1 | serine/threonine protein kinase, FIKK family | 58T>C | Tyr20His |
| **PF3D7_0902500** | 9:109334-111329 | 1 | serine/threonine protein kinase, FIKK family | 865T>C | Cys289Arg |
| **PF3D7_0903300** | 9:140977-150588 | 2 | conserved Plasmodium membrane protein, unknown function | 2518A>T; 4167A>T | Ile840Phe; Lys1389Asn |
| **PF3D7_0904200** | 9:197867-198816 | 1 | PH domain-containing protein, putative | 465C>A | Asn155Lys |
| **PF3D7_0904300** | 9:199051-207458 | 1 | conserved protein, unknown function | 3445T>A | Tyr1149Asn |
| **PF3D7_0904600** | 9:212515-217983 | 1 | ubiquitin specific protease, putative | 2032A>G | Ile678Val |
| **PF3D7_0905300** | 9:251353-269709 | 1 | dynein heavy chain, putative | 2182A>T | Asn728Tyr |
| **PF3D7_0905500** | 9:278369-279088 | 1 | conserved Plasmodium protein, unknown function | 65C>G | Ala22Gly |
| **PF3D7_1001600** | 10:86538-89009 | 1 | alpha/beta hydrolase, putative | 1676C>T | Ala559Val |
| **PF3D7_1004400** | 10:207000-209642 | 2 | RNA-binding protein, putative | 776C>G; 575A>G | Ala259Gly; Asn192Ser |
| **PF3D7_1005300** | 10:233285-234473 | 2 | conserved Plasmodium protein, unknown function | 759A>T; 11A>T | Glu253Asp; Asn4Ile |
| **PF3D7_1005400** | 10:234795-235745 | 1 | conserved Plasmodium protein, unknown function | 225T>A | Asn75Lys |
| **PF3D7_1107300** | 11:301283-311287 | 1 | polyadenylate-binding protein-interacting protein 1, putative | 9430A>T | Ile3144Phe |
| **PF3D7_1129300** | 11:1131294-1136834 | 1 | conserved Plasmodium protein, unknown function | 1477G>A | Gly493Arg |
| **PF3D7_1138400** | 11:1501011-1513691 | 1 | guanylyl cyclase | 11060A>G | Tyr3687Cys |
| **PF3D7_1139100** | 11:1548186-1553561 | 1 | RNA-binding protein, putative | 4289G>A | Arg1430Lys |
| **PF3D7_1139300** | 11:1556744-1565045 | 3 | transcription factor with AP2 domain(s) | 7404A>C; 5701G>A; 1577C>G | Gln2468His; Gly1901Ser; Thr526Ser |
| **PF3D7_1342200** | 13:1660812-1663730 | 1 | conserved Plasmodium membrane protein, unknown function | 2902G>A | Asp968Asn |
| **PF3D7_1343700** | 13:1724817-1726997 | 1 | kelch protein K13 | 566A>C | Lys189Thr |
| **PF3D7_1344000** | 13:1759466-1761991 | 1 | aminomethyltransferase, putative | 922A>G | Lys308Glu |
| **PF3D7_1344100** | 13:1764190-1766607 | 1 | krox-like protein, putative | 2170C>G | Gln724Glu |
| **PF3D7_1346400** | 13:1852898-1870864 | 1 | VPS13 domain-containing protein, putative | 9661C>A | His3221Asn |
| **PF3D7_1346700** | 13:1876016-1877362 | 2 | 6-cysteine protein | 1307C>T; 940T>A | Thr436Ile; Leu314Ile |
| **PF3D7_1346800** | 13:1878875-1880194 | 2 | 6-cysteine protein | 532A>G; 203C>T | Ile178Val; Thr68Met |
| **PF3D7_1360800** | 13:2435343-2438924 | 1 | falcilysin | 28A>T | Ile10Phe |
| **PF3D7_1417700** | 14:750330-751684 | 1 | conserved Plasmodium protein, unknown function | 364G>T | Asp122Tyr |
| **PF3D7_1418900** | 14:783078-785819 | 1 | ATP-dependent RNA helicase DBP4, putative | 2135T>C | Val712Ala |
| **PF3D7_1419400** | 14:804425-811369 | 3 | conserved Plasmodium membrane protein, unknown function | 5150G>T; 2898T>A; 2605G>A | Gly1717Val; Asn966Lys; Asp869Asn |
| **PF3D7_1453600** | 14:2199371-2204708 | 2 | RAP protein, putative | 3689A>T; 3669T>G | Lys1230Ile; Asn1223Lys |
| **PF3D7_1474200** | 14:3023852-3038832 | 1 | conserved Plasmodium membrane protein, unknown function | 9554C>T | Ser3185Leu |
| **PF3D7_1478100** | 14:3216275-3217794 | 1 | Plasmodium exported protein (hyp13), unknown function | 721A>G | Ile241Val |

Table C - Significant NSY mutations associated with clone persistence (within MAF = 0.46)

| Position | Gene | days | Location | Description |
| --- | --- | --- | --- | --- |
| 1_532799 | PF3D7_0113800 | 150.4566 | 1:527107-536351 | DBL containing protein, unknown function |
| 4_933387 | PF3D7_0420600 | 149.84 | 4:932463-934244 | conserved Plasmodium protein, unknown function |
| 6_340576 | PF3D7_0608100 | 183.0331 | 6:337792-341760 | conserved Plasmodium protein, unknown function |
| 7_1055227 | PF3D7_0724900 | 147.7724 | 7:1052465-1058158 | kinesin-19, putative |
| 8_129755 | PF3D7_0801900 | 162.3918 | 8:128849-142837 | conserved Plasmodium protein, unknown function |
| 8_129758 | PF3D7_0801900 | 164.3136 | 8:128849-142837 | conserved Plasmodium protein, unknown function |
| 10_1391602 | PF3D7_1035100 | 148.887 | 10:1391445-1393130 | probable protein, unknown function |
| 11_265310 | PF3D7_1106300 | 154.6216 | 11:263604-266570 | exonuclease, putative |
| 11_1295197 | PF3D7_1133400 | 178.7863 | 11:1293856-1295724 | apical membrane antigen 1 |
| 11_1295605 | PF3D7_1133400 | 171.3212 | 11:1293856-1295724 | apical membrane antigen 1 |
| 12_63352 | PF3D7_1200700 | 148.0282 | 12:61556-64336 | acyl-CoA synthetase |
| 12_2145292 | PF3D7_1252500 | 151.1277 | 12:2145266-2146616 | Plasmodium exported protein, unknown function |
| 13_1074596 | PF3D7_1325800 | 187.08 | 13:1071146-1075747 | conserved Plasmodium protein, unknown function |
| 13_1074599 | PF3D7_1325800 | 187.08 | 13:1071146-1075747 | conserved Plasmodium protein, unknown function |
| 13_1074605 | PF3D7_1325800 | 186.1493 | 13:1071146-1075747 | conserved Plasmodium protein, unknown function |
| 13_1074611 | PF3D7_1325800 | 187.08 | 13:1071146-1075747 | conserved Plasmodium protein, unknown function |
| 13_2435370 | PF3D7_1360800 | 155.8896 | 13:2435343-2438924 | falcilysin |
| 14_621244 | PF3D7_1415400 | 167.426 | 14:618897-623321 | conserved Plasmodium protein, unknown function |
| 14_621249 | PF3D7_1415400 | 165.6374 | 14:618897-623321 | conserved Plasmodium protein, unknown function |
| 14_621254 | PF3D7_1415400 | 167.426 | 14:618897-623321 | conserved Plasmodium protein, unknown function |
| 14_621268 | PF3D7_1415400 | 167.426 | 14:618897-623321 | conserved Plasmodium protein, unknown function |
| 14_2354379 | PF3D7_1457400 | 151.1176 | 14:2350985-2360152 | conserved Plasmodium protein, unknown function |
| 14_2356794 | PF3D7_1457400 | 154.4295 | 14:2350985-2360152 | conserved Plasmodium protein, unknown function |
| 14_2369196 | PF3D7_1457700 | 148.3871 | 14:2367287-2371723 | large ribosomal subunit nuclear export factor,putative |

Table D - Significant NSY mutations associated with clonal abundance (within MAF = 0.46)

| Position | Gene | days | Location | Description |
| --- | --- | --- | --- | --- |
| 1_500968 | PF3D7_0113200 | 4.4737 | 1:500959-502868 | Plasmodium exported protein, unknown function |
| 5_510453 | PF3D7_0511500 | 4.2517 | 5:486602-516787 | RNA pseudouridylate synthase, putative |
| 6_340576 | PF3D7_0608100 | 5.3974 | 6:337792-341760 | conserved Plasmodium protein, unknown function |
| 9_1205332 | PF3D7_0930300 | 4.2889 | 9:1201812-1206974 | merozoite surface protein 1 |
| 11_265310 | PF3D7_1106300 | 4.3446 | 11:263604-266570 | exonuclease, putative |
| 11_1295197 | PF3D7_1133400 | 4.6031 | 11:1293856-1295724 | apical membrane antigen 1 |
| 11_1295605 | PF3D7_1133400 | 4.4234 | 11:1293856-1295724 | apical membrane antigen 1 |
| 13_1074596 | PF3D7_1325800 | 5.215 | 13:1071146-1075747 | conserved Plasmodium protein, unknown function |
| 13_1074599 | PF3D7_1325800 | 5.215 | 13:1071146-1075747 | conserved Plasmodium protein, unknown function |
| 13_1074605 | PF3D7_1325800 | 5.199 | 13:1071146-1075747 | conserved Plasmodium protein, unknown function |
| 13_1074611 | PF3D7_1325800 | 5.22 | 13:1071146-1075747 | conserved Plasmodium protein, unknown function |
| 13_2193129 | PF3D7_1355200 | 4.272 | 13:2191058-2194111 | RAP protein, putative |
| 14_621244 | PF3D7_1415400 | 4.4438 | 14:618897-623321 | conserved Plasmodium protein, unknown function |
| 14_621249 | PF3D7_1415400 | 4.4094 | 14:618897-623321 | conserved Plasmodium protein, unknown function |
| 14_621254 | PF3D7_1415400 | 4.4438 | 14:618897-623321 | conserved Plasmodium protein, unknown function |
| 14_621268 | PF3D7_1415400 | 4.4438 | 14:618897-623321 | conserved Plasmodium protein, unknown function |
| 14_2999453 | PF3D7_1473700 | 4.4701 | 14:2998594-3002355 | nucleoporin NUP116/NSP116, putative |

Table E - Genes identify in isoRelate for 2016-2017 (n=24)

| Gene | location | function |
| --- | --- | --- |
| PF3D7_0603000 | 6:122634124412 | conserved Plasmodium protein, unknown function |
| PF3D7_0708500 | 7:385583388321 | heat shock protein 86 family protein |
| PF3D7_0710200 | 7:463105471837 | conserved Plasmodium protein, unknown function |
| PF3D7_0711400 | 7:500408502594 | histone deacetylase complex subunit SAP18, putative |
| PF3D7_0711500 | 7:505245507311 | regulator of chromosome condensation, putative |
| PF3D7_0711600 | 7:508474508609 | Plasmodium RNA of unknown function RUF61 |
| PF3D7_0713200 | 7:607111607785 | RESA-like protein |
| PF3D7_0713300 | 7:609297610522 | erythrocyte membrane protein 1 (PfEMP1), pseudogene |
| PF3D7_0713400 | 7:612458613740 | serpentine receptor, putative |
| PF3D7_0713500 | 7:614385617249 | conserved Plasmodium protein, unknown function |
| PF3D7_0713600 | 7:617933620867 | mitochondrial ribosomal protein S5 precursor, putative |
| PF3D7_0713700 | 7:621463623521 | conserved Plasmodium protein, unknown function |
| PF3D7_0713900 | 7:627067637776 | conserved Plasmodium protein, unknown function |
| PF3D7_0715200 | 7:676061682681 | conserved Plasmodium protein, unknown function |
| PF3D7_0715900 | 7:699239700909 | zinc transporter, putative |
| PF3D7_0715800 | 7:695600696904 | drug/metabolite exporter, drug/metabolite transporter |
| PF3D7_0716000 | 7:703207706584 | RNA binding protein, putative |
| PF3D7_0716200 | 7:712469714967 | conserved Plasmodium protein, unknown function |
| PF3D7_0716100 | 7:707699710908 | protein SDA1, putative |
| PF3D7_0827200 | 8:11773101180324 | conserved Plasmodium protein, unknown function |
| PF3D7_1034600 | 10:13768271377759 | translation initiation factor IF3, putative |
| PF3D7_1034900 | 10:13834501386119 | methioninet RNA ligase |
| PF3D7_1034800 | 10:13800281382249 | conserved Plasmodium protein, unknown function |
| PF3D7_1035000 | 10:13866491389630 | U2 snRNA/tRNA pseudouridine synthase, putative |

Table F - Genes identify in isoRelate for 2020-2021 (n=16)

| Gene | location | function |
| --- | --- | --- |
| PF3D7_0402000 | 4:114601116058 | Plasmodium exported protein (PHISTa), unknown function |
| PF3D7_0413900 | 4:623168626226 | ubiquitin carboxylterminal hydrolase 13,putative |
| PF3D7_0414100 | 4:634029638905 | conserved Plasmodium membrane protein, unknown function |
| PF3D7_0421700 | 4:987842994360 | conserved Plasmodium protein, unknown function |
| PF3D7_0619800 | 6:831286839033 | conserved Plasmodium membrane protein, unknown function |
| PF3D7_0710200 | 7:463105471837 | conserved Plasmodium protein, unknown function |
| PF3D7_0710500 | 7:476116477552 | conserved Plasmodium protein, unknown function |
| PF3D7_0710300 | 7:472501473541 | conserved Plasmodium membrane protein, unknown function |
| PF3D7_0710700 | 7:481369481974 | conserved Plasmodium protein, unknown function |
| PF3D7_0903300 | 9:140977150588 | conserved Plasmodium membrane protein, unknown function |
| PF3D7_0903400 | 9:151700159541 | DEAD/DEAH box helicase, putative |
| PF3D7_0903500 | 9:162769166923 | conserved Plasmodium protein, unknown function |
| PF3D7_0903800 | 9:179187183991 | LCCL domaincontaining protein |
| PF3D7_0904100 | 9:190858195827 | AP4 complex subunit epsilon, putative |
| PF3D7_0929400 | 9:11752031180762 | high molecular weight rhoptry protein 2 |
| PF3D7_1003400 | 10:152364158516 | conserved Plasmodium protein, unknown function |

Table G – Prevalence of C350R among the different genomic clusters

| **Cluster** | Isolates (2016-17) | Total isolates (2016-17) | % with C350R (2016-17) | Isolates (2020) | Total isolates (2020-21) | % with C350R (2010-21) |
| --- | --- | --- | --- | --- | --- | --- |
| 1 | 78 | 80 | 97.5 | 1 | 2 | 50.0 |
| **2** | 92 | 92 | 100.0 | 20 | 38 | 52.6 |
| **3** | 1 | 18 | 5.6 | 15 | 105 | 14.3 |
| **4** | 5 | 5 | 100.0 | 43 | 61 | 70.5 |
| **5** | 52 | 53 | 98.1 | - | - | - |
| 6 | 18 | 18 | 100.0 | 0 | 36 | 0.0 |
| **7** | 64 | 112 | 57.1 | 27 | 46 | 58.7 |
| **8** | 42 | 49 | 85.7 | 13 | 13 | 100.0 |
| **9** | 1 | 2 | 50.0 | 37 | 39 | 94.9 |
| **10** | 0 | 13 | 0.0 | 0 | 2 | 0.0 |
| **11** | 1 | 8 | 12.5 | 4 | 32 | 12.5 |
| **12** | 40 | 51 | 78.4 | 0 | 2 | 0.0 |
| **13** | 20 | 44 | 45.5 | 7 | 34 | 20.6 |
| **Other** | 64 | 107 | 59.8 | 24 | 118 | 20.3 |
| **Total** | 478 | 652 | 73.3 | 191 | 528 | 36.2 |

Table H – Epidemiological zones associated with sample provenance

| Epidemiological zone | Highly related cluster | Communities | Isolates | Isolates (%) |
| --- | --- | --- | --- | --- |
| **Lower Mazaruni** | 13 | 151 | 434 | 36% |
| **Potaro** | 11 | 53 | 162 | 13% |
| **Lower Cuyuni** | 12 | 62 | 142 | 12% |
| **Upper Cuyuni** | 11 | 38 | 95 | 8% |
| **Kaituma and Barima** | 12 | 29 | 66 | 5% |
| **Lower Essequibo** | 8 | 27 | 50 | 4% |
| **Mid Mazaruni** | 10 | 19 | 38 | 3% |
| **Upper Mazaruni** | 10 | 16 | 31 | 3% |
| **Mid Essequibo** | 8 | 27 | 56 | 5% |
| **Waini** | 7 | 15 | 29 | 2% |
| **Greater GT** | 7 | 14 | 24 | 2% |
| **Central Coast** | 9 | 11 | 23 | 2% |
| **Cristinas Border** | 6 | 10 | 14 | 1% |
| **North Delta** | 6 | 10 | 13 | 1% |
| **Head Mazaruni** | 5 | 9 | 10 | 1% |
| **West of GT** | 5 | 5 | 5 | 0% |
| **Demerara** | 5 | 5 | 6 | 0% |
| **East of GT** | 2 | 2 | 2 | 0% |
| **Chenapau** | 2 | 2 | 2 | 0% |
| **Greater Lethem** | 1 | 1 | 1 | 0% |
| **TOTAL** |  |  | 1203 | 100% |

# Supplementary Figures

Fig A – IBD distribution in the dataset (1,445 genomes)
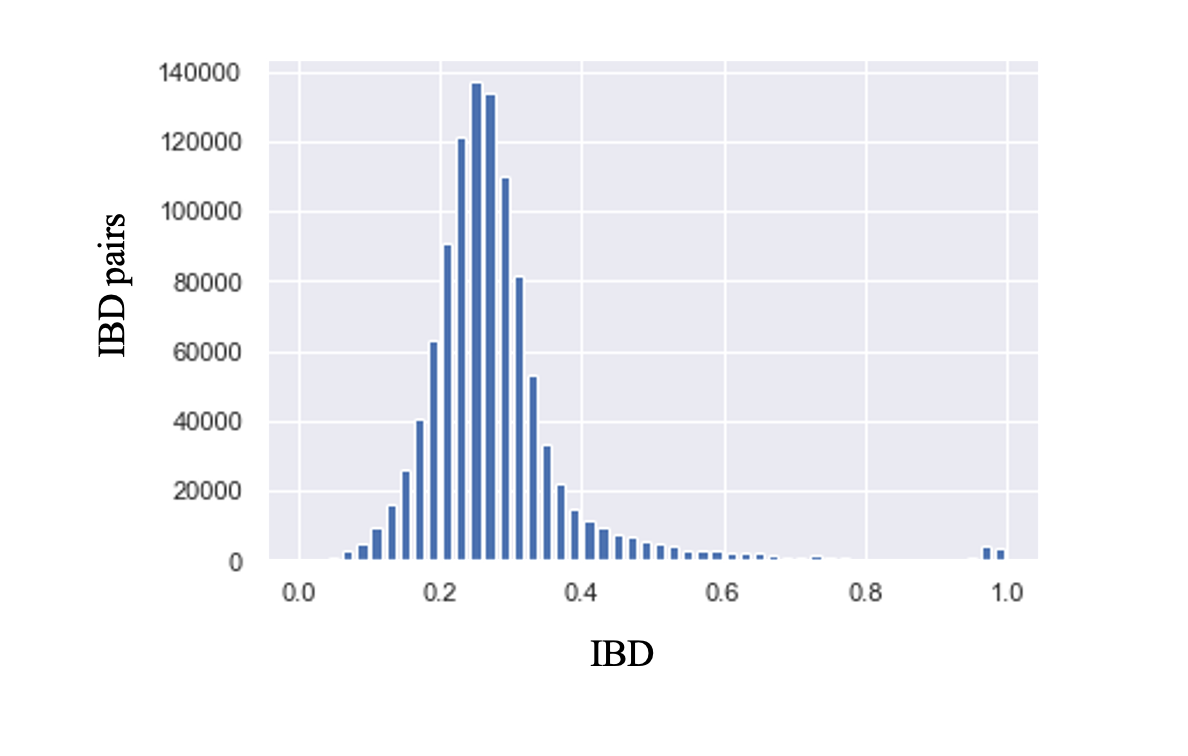


Fig B – Average coverage using an overlapping sliding window (size =1000, overlap=100) for the samples sequenced in two batches: the isolates obtained over the 2016-2019 period and the 2020-2021 period.


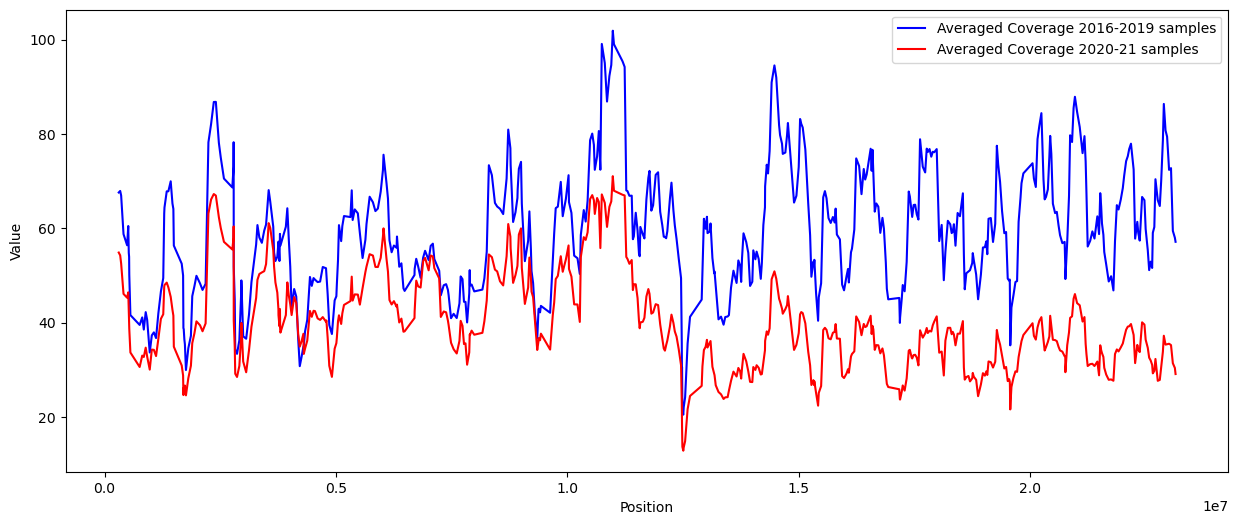


Fig C - The mean IBD between samples highlighting highly related cluster 1 - (n=107 isolates)


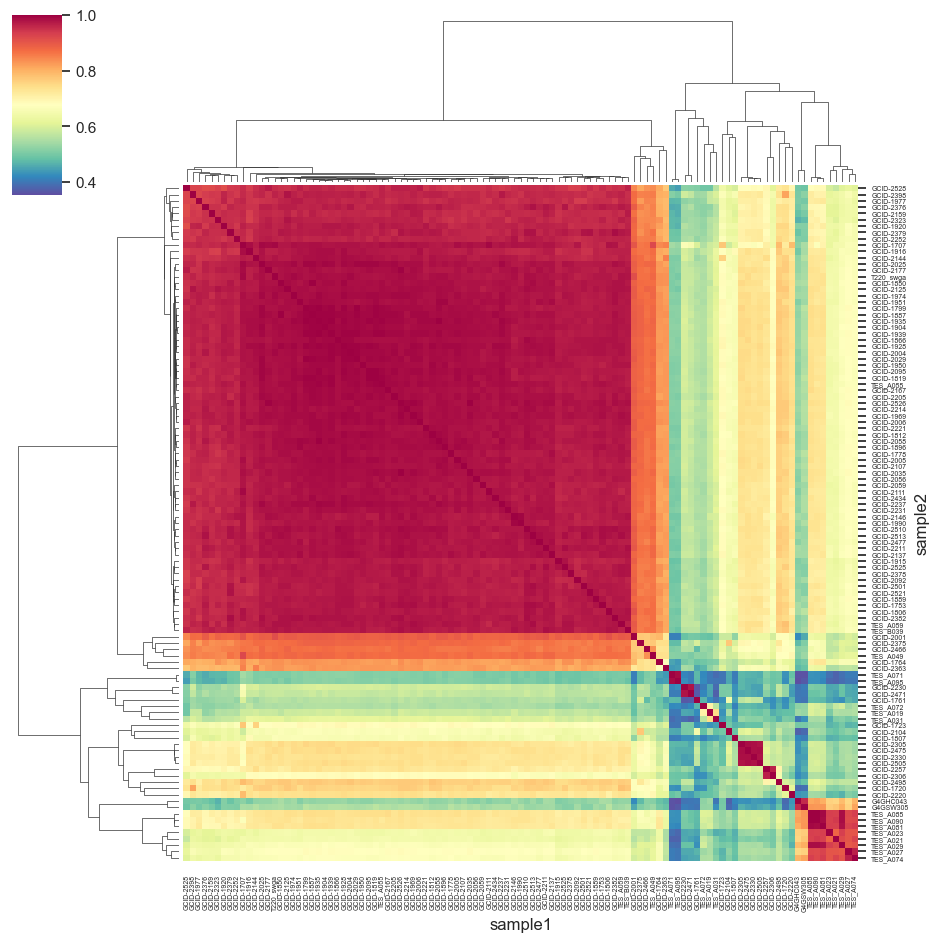


Fig D – Country-wide distribution of highly related clusters. Map was created using Basemap v1.4.1. plotted using Guyana - Subnational Administrative Boundaries acquired from [75].


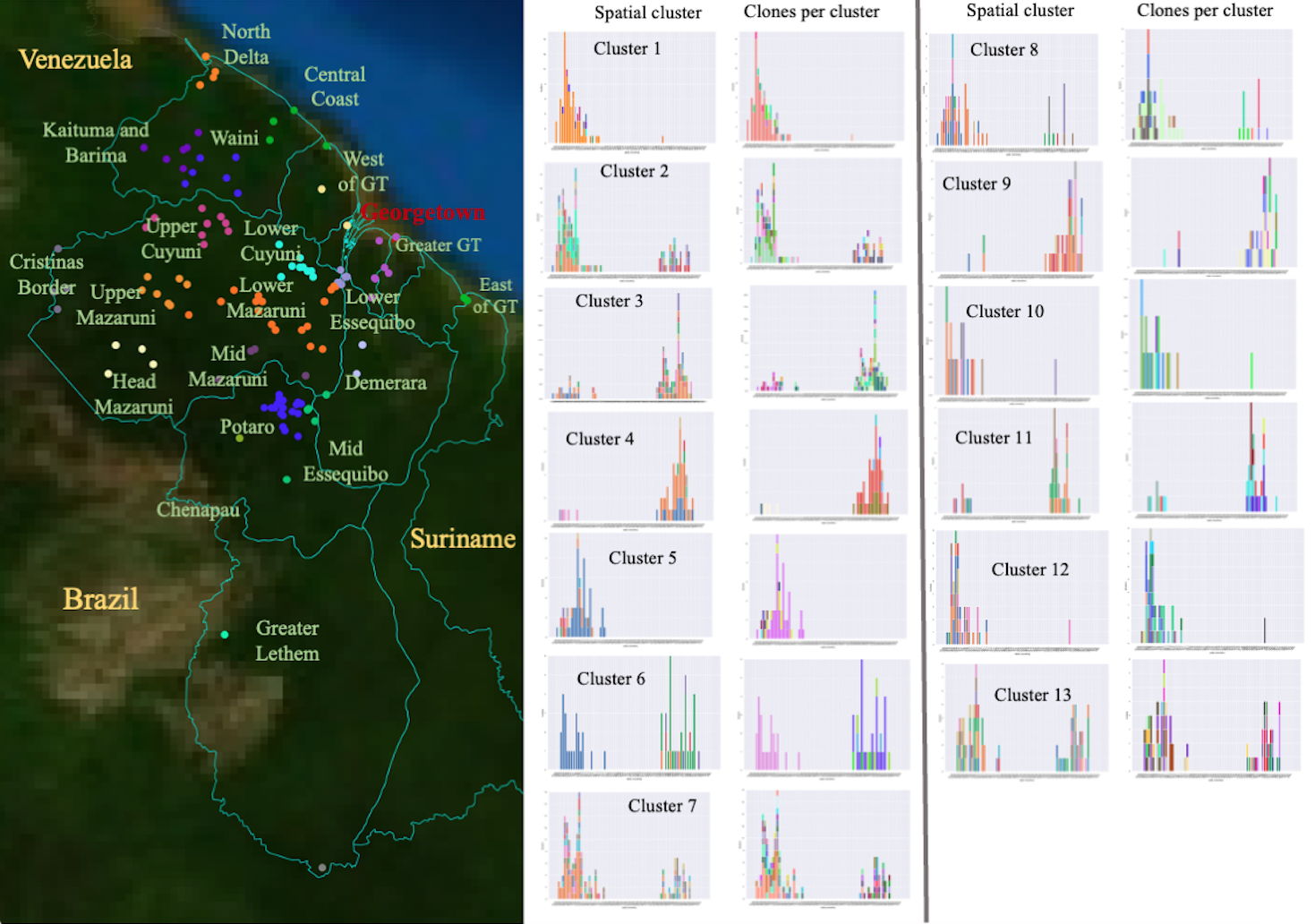


Fig E – Relatedness for the two study periods – 2016-2017 and 2020-2021


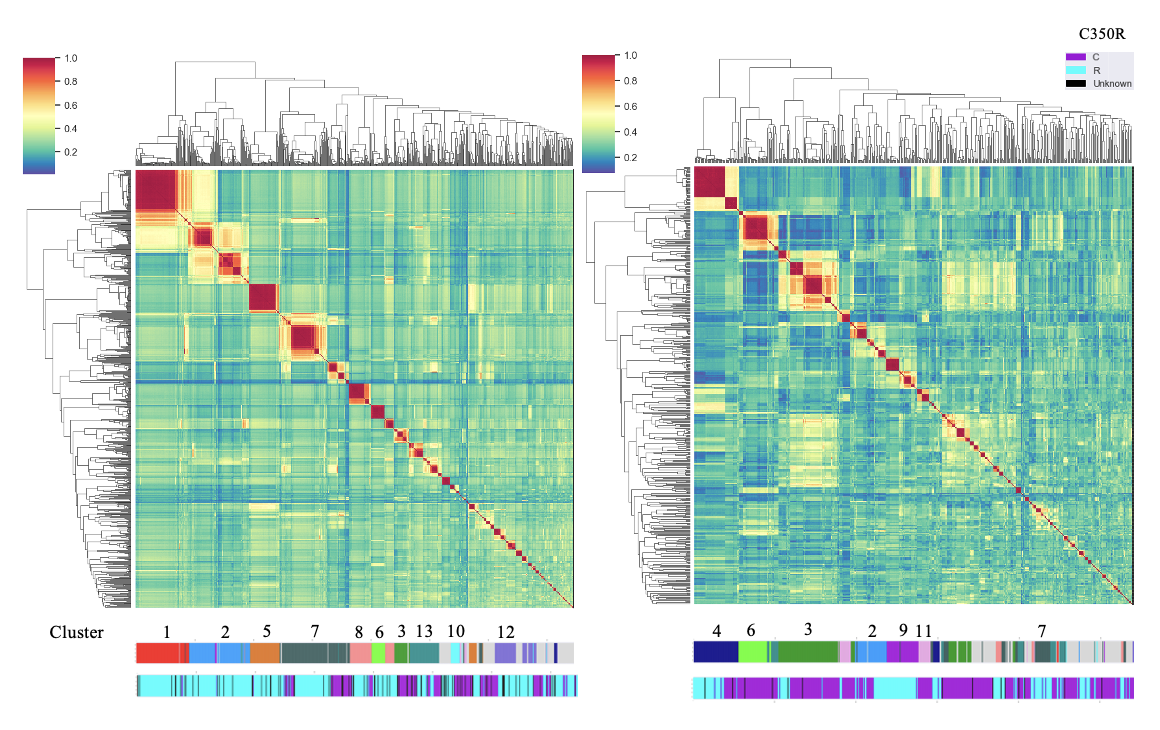


Fig F – Change mutation frequencies across 2016-2017 and 2020-2021


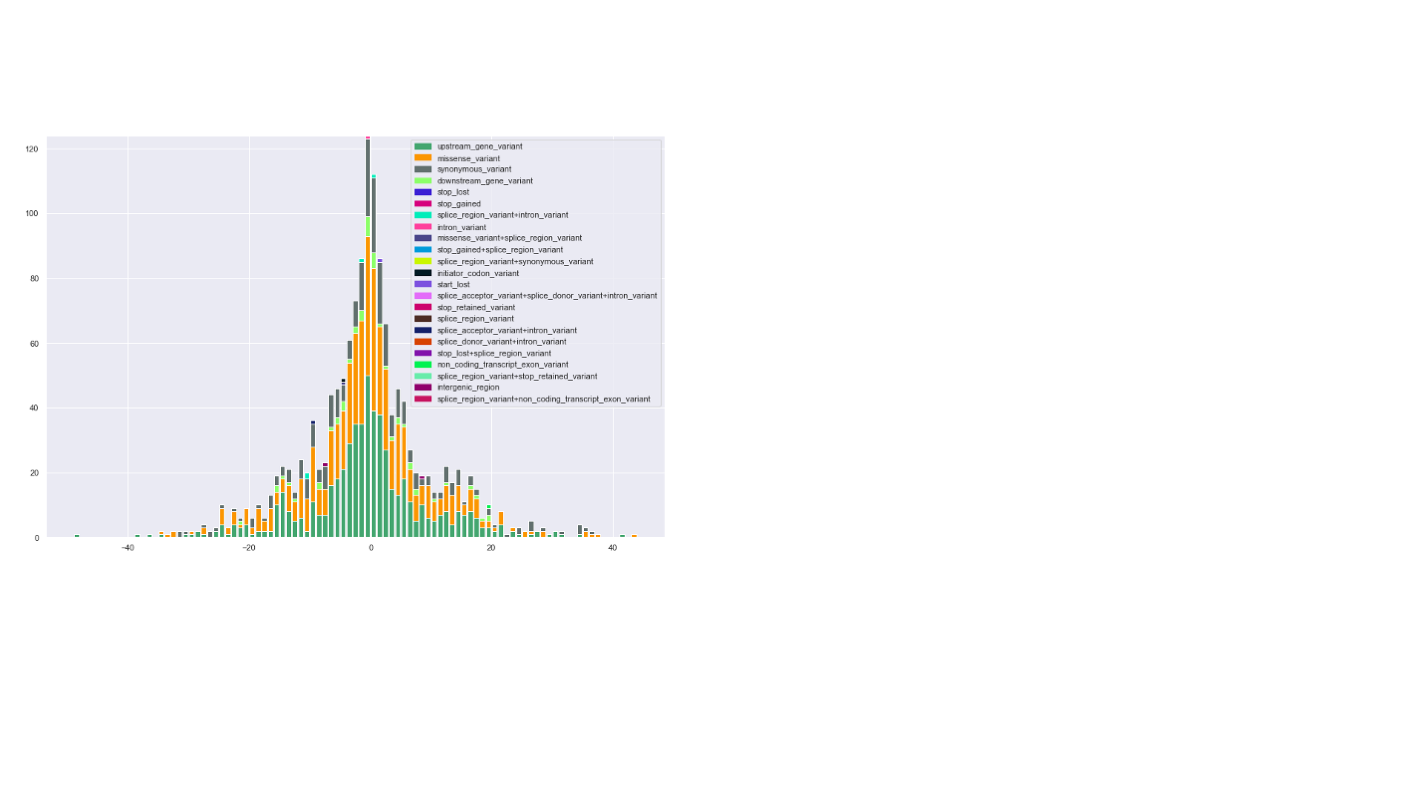


Fig G – Haplotypes in a 10kb window for four different genes. Rows represent individual genome and columns are alleles: mutants are colored in green while wiltype is in white, grey represents missing data.


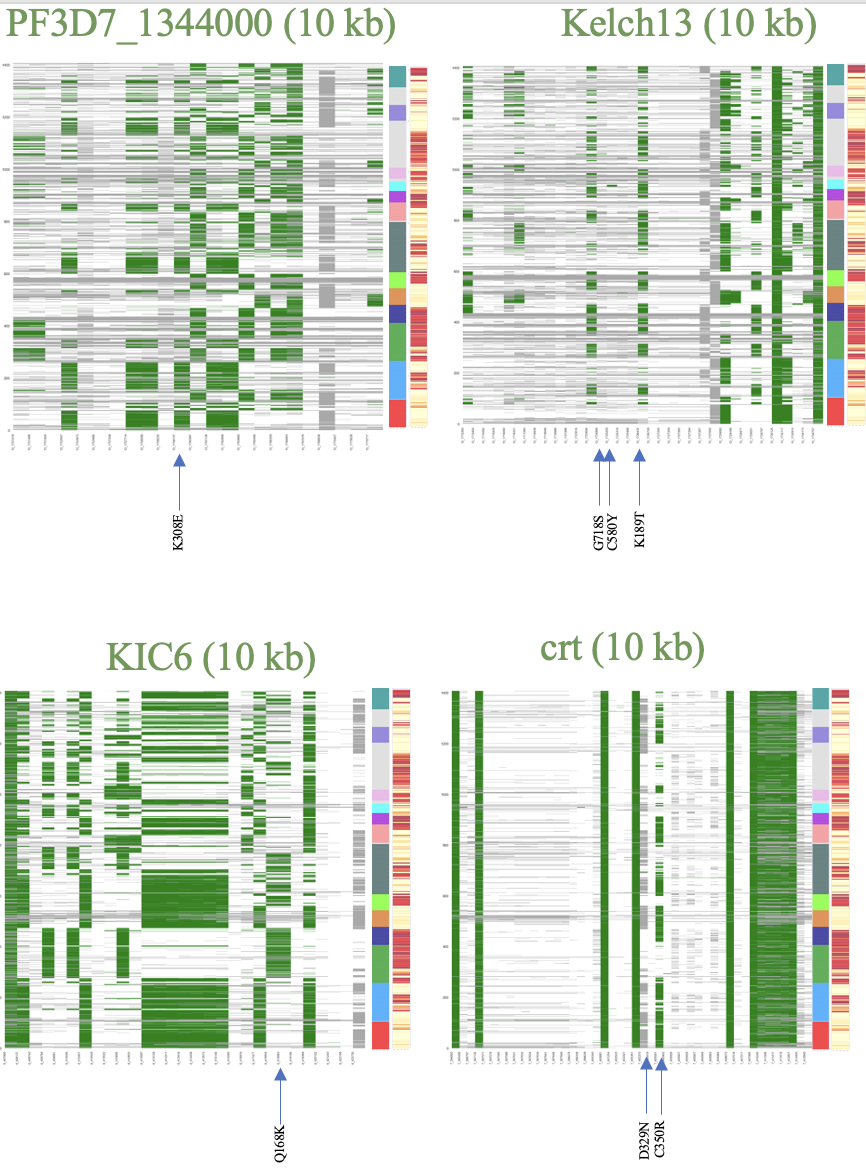


Fig H – Selection signals from isoRelate in clones and singleton isolate sampled over two study periods (a-b) 2016-2017 and (c-d) 2020-2021


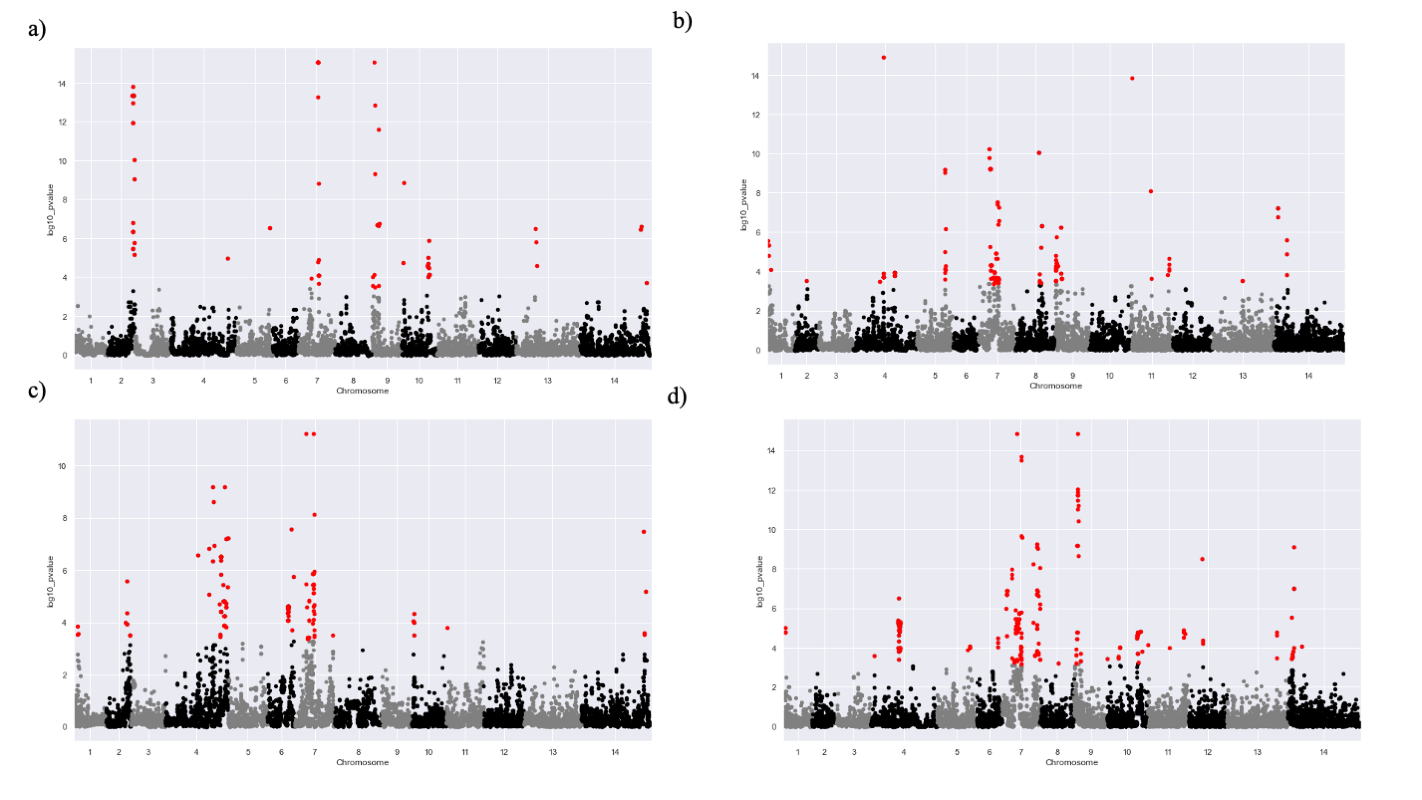


Fig I – Selection signals from isoRelate between clones carrying *pfcrt* C350R and wildtype clones.


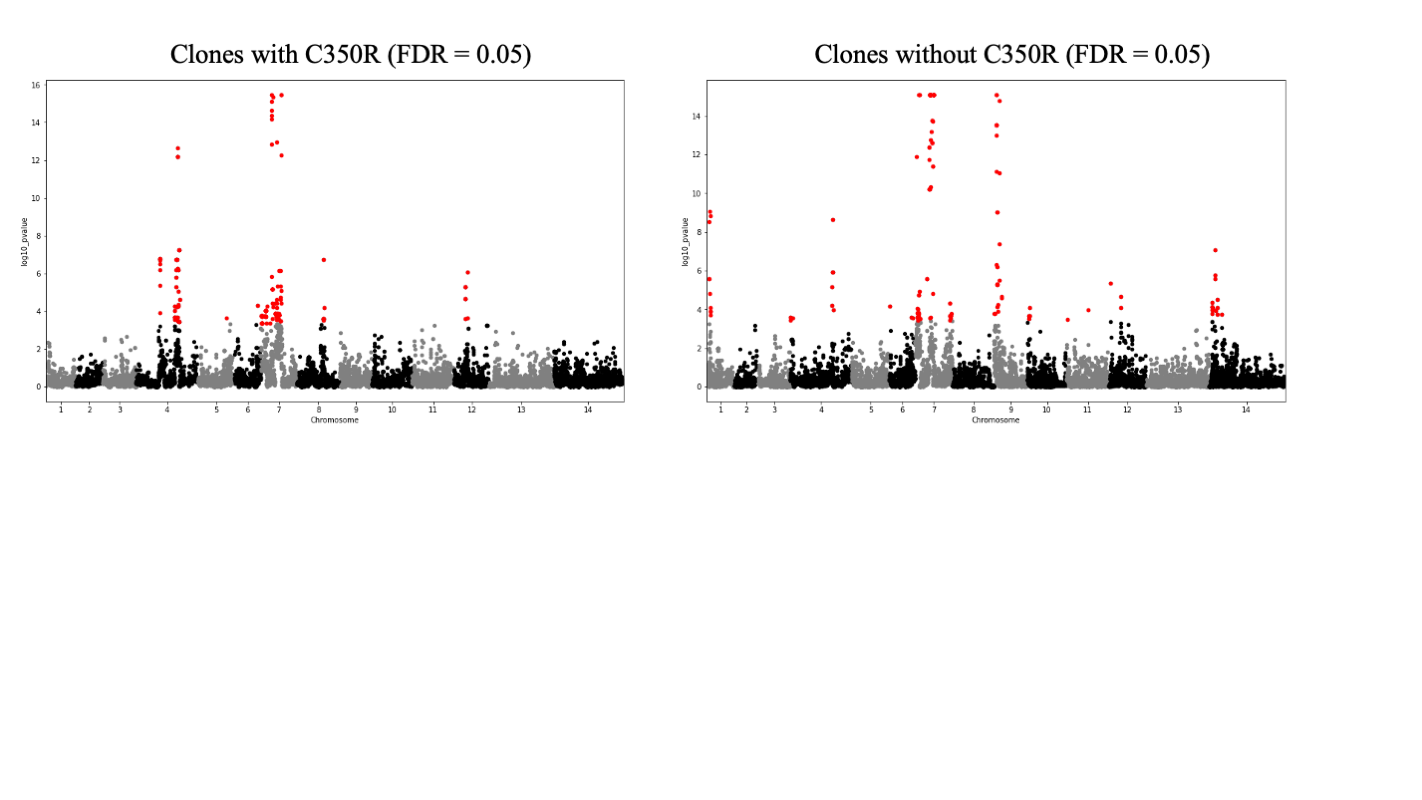


Fig J – Mean pairwise IBD within 100-kb overlapping windows with 10-kb overlap different chromosomes for long-lasting and short lasting clones over the two time periods. a-d) isoRelate results for the different datasets, e-f) mean pairwise IBD on chromosome 7, i-l) mean pairwise IBD on chromosome 9


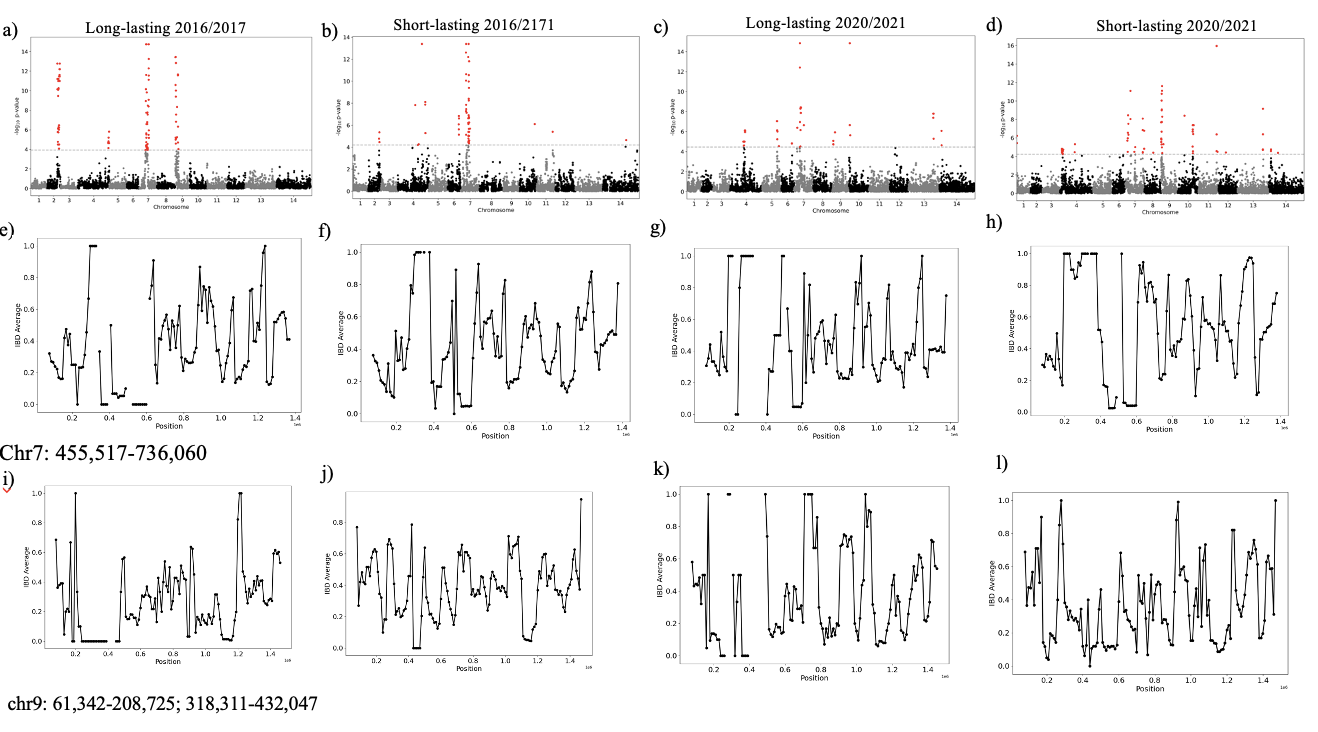


Fig K – Mean pairwise IBD within 100-kb overlapping windows with 10-kb overlap different chromosomes for long-lasting and short lasting clones over the two time periods. a-d) isoRelate results for the different datasets, e-f) mean pairwise IBD on chromosome 2, i-l) mean pairwise IBD on chromosome 4


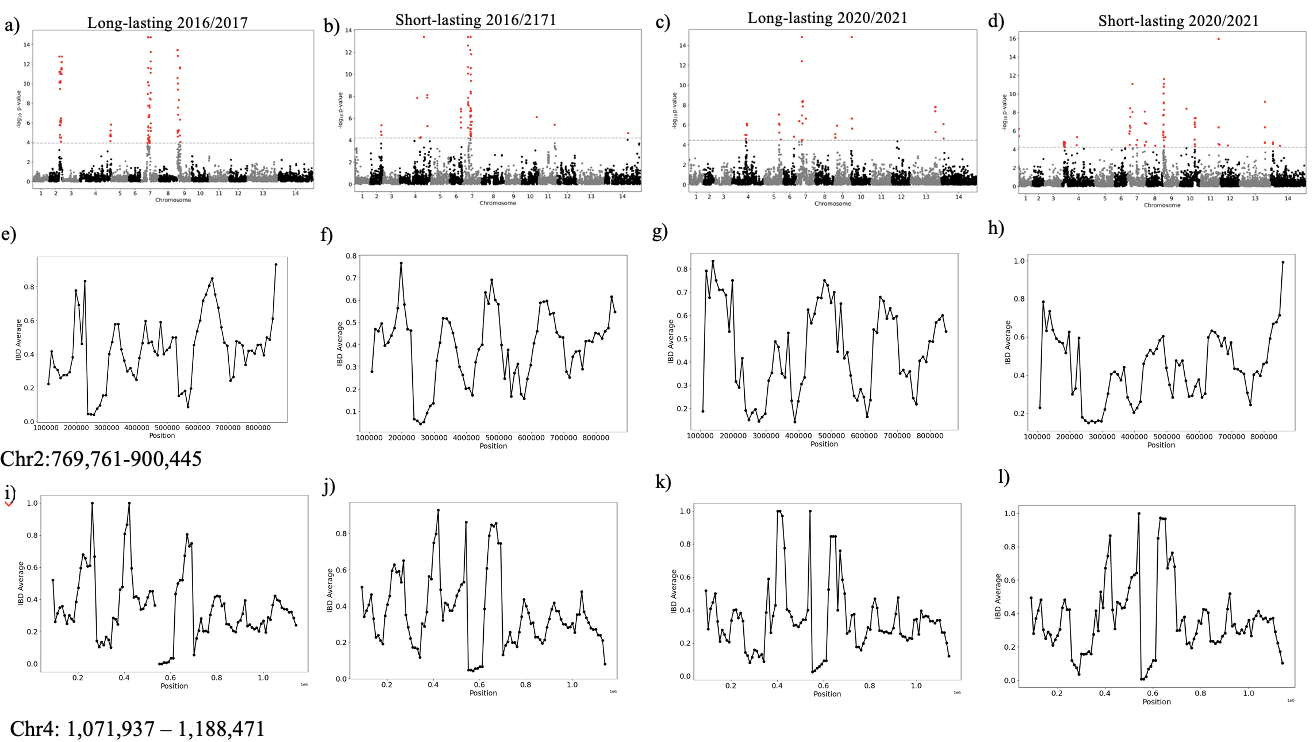

Supplement: S1 Text — Table A. Emerging highly related clusters emerging in Guyana. Highly related clusters are defined as a group of at least 3 clones with an average IBD ≥ 0.40. Table B. Polymorphism which increased in frequency between 2016–2017 and 2020–2021. Table C. Significant NSY mutations associated with clone persistence (within MAF = 0.46). Table D. Significant NSY mutations associated with clonal abundance (within MAF = 0.46). Table E. Genes identify in isoRelate for 2016–2017 (n = 24). Table F. Genes identify in isoRelate for 2020–2021 (n = 16). Table G. Prevalence of C350R among the different genomic clusters. Table H. Epidemiological zones associated with sample provenance. Fig A. IBD distribution in the dataset (1,445 genomes). Fig B. Average coverage using an overlapping sliding window (size = 1000, overlap = 100) for the samples sequenced in two batches: the isolates obtained over the 2016–2019 period and the 2020–2021 period. Fig C. The mean IBD between samples highlighting highly related cluster 1—(n = 107 isolates). Fig D. Country-wide distribution of highly related clusters. Fig E. Relatedness for the two study periods– 2016–2017 and 2020–2021. Fig F. Change mutation frequencies across 2016–2017 and 2020–2021. Fig G. Haplotypes in a 10kb window for four different genes. Rows represent individual genome and columns are alleles: mutants are colored in green while wildtype is in white, grey represents missing data. Fig H. Selection signals from isoRelate in clones and singleton isolate sampled over two study periods (a-b) 2016–2017 and (c-d) 2020–2021. Fig I. Selection signals from isoRelate between clones carrying pfcrt C350R and wildtype clones. Fig J. Mean pairwise IBD within 100-kb overlapping windows with 10-kb overlap different chromosomes for long-lasting and short lasting clones over the two time periods. a-d) isoRelate results for the different datasets, e-f) mean pairwise IBD on chromosome 7, i-l) mean pairwise IBD on chromosome 9. Fig K. Mean pairwise IBD wi [file ppat.1012013.s002.docx]
