# Supplementary material for: Temporal and spatial dynamics of Plasmodium falciparum clonal lineages in Guyana
Source: PLoS Pathog. 2024 Jun 13;20(6):e1012013. doi: 10.1371/journal.ppat.1012013 (PMC11206942; doi:10.1371/journal.ppat.1012013)
Supplement: Attachment — Submitted filename: Response to reviewers.pdf [file ppat.1012013.s004.pdf]

Response to reviewers

>>> We thank the reviewers and the editor for providing very helpful comments. We hope the reviewers and editor will find that our responses to the comments make the manuscript more readable. Generally, we have attempted to make the results easier to follow and clarified the figure as suggested by the reviewers. Please note that line numbers referenced below correspond to the manuscript draft with changes tracked.

Reviewer #1

1. Data were not presented sequentially and were hard to follow. For example, in line 194ff, the authors described that C#134 was found in Mid Essenquibo but also found in other spatial clusters (Fig. 3). But Fig 3 illustrated the temporal dynamics of the genetically related clusters. Then, the authors mentioned in line 196ff that C#137 occurred in 9 spatial clusters (Fig. 6) (Same for line 199). But Fig 6 shows selection signals on different chromosomes. The data need to be presented sequentially, not jumping from fig 3 to fig 6. Then, the authors presented data for Fig 7 (line 213ff), and finally touched upon the selection landscape (Fig. 6, line 249ff).

>>> We made sure the figures are now presented sequentially and therefore reordered some of the figures. We also reviewed the relevance of reference to figure in the text. For instance, in L209, the C#134's spatial location now only points to Fig. S4 and C#137 occurred in nine spatial clusters (now referred to as epidemiological zones - Fig. 5 - L213).

2. Figure 1. Please use a white background to mark the sites, since the color scheme in a dark green background is difficult to view. There are also samples marked in black in Fig 1b – do these samples also represent the ones without traveling histories? Where were the samples collected to document the travel histories?

>>> The Fig. 1a has been updated varying the opacity of the background (alpha 0.85). On Fig. 1b, samples with no traveling histories are indeed in black, this has been noted on the figure.

3. While Figure 2 provides a holistic view of the clusters, it would be more illustrative if the authors could use cluster 1 as an example to make a separate cladogram to show the relationships of the different clonal populations and the singletons within this cluster.

>>> This has been added in the supplement section (Fig. S3) and was added in the text L158.

4. Figure 3: The labeling of clusters 1-13 on the left of the figure makes it hard to see which parasites are referred. Could you use a "{" sign to clearly mark the boundary of each cluster?

>>> To highlight the boundary of the different clusters, we now added a transparent colored background which allows the reader to better visualize the duration and extent of each of the different clusters.

5. Figure 4 – hard to view and understand: many colors that differ only slightly and overlap extensively make it difficult to follow. Could the authors present a more concise illustration of the main clusters here? Also, please explain what the density figure shows.

>>> This is true, however, the bulk of the clones (highlighted by the density plot) is not the main focus of the figure. We tried to show that among these clones, some happened to have traveled to more epidemiological zones than others. A sentence was added to explain the presence of the density plot.

6. Line 175: For those who don't know the geography of Guyana, it is difficult to figure out where Aranka River in Region 7 is located. Please refer to a closer site that is labeled in figure 1.

>>> We added the region number on Fig. 1.

7. Line 225: Highly related clusters with more than 2 samples displayed a frequency of pfpm2/3 copy number variation of 37.8% - what does this mean?

>>> Fig. 6 displays the presence of *pfcrt* C350R and *pfpm2/3* copy number variation among clones and we agree that the sentence was ambiguous. The sentence now reads "Among clones tested for the presence of *pfpm2/3* copy number variation (CNV), the frequency of *pfpm2/3* CNV was 37.8% (Fig. 6). " (L245-L247).

8. Mutations in drug resistance genes (237ff): in this section, mutations were found in # samples – also provide the % (as in line 240). For all the mutations, please also mention the amino acids for the positions (for example: 350R, like line 246 – G442H).

>>> These changes have been implemented throughout the paragraph (L262; L263; L264; L266; L269, L270) as well as in the following one (L282; L284; 289; L290).

9. Selection landscape (line 249ff): The authors provided many details on the genes with increased frequencies in the two study periods. Given that the sampling was not systematic, many samples were clonal, mutations may result from stochastic processes instead of drug selection, and none of them were validated genetically, I think this section should be concise without too much speculation. I think Table 2 should be moved to supplement, as it contains a long list of genes, many of which have no known functions or are not linked to drug resistance.

>>> We agree that Table 2 takes a significant amount of space and we followed this advice and moved it into the supplement, replacing Table S2.

10. Table 1 needs to be cited in results – when the 13 clusters were analyzed. The first time I see this Table was in the Discussion (line 309).

>>> This is true, the reference to Table 1 has been added in the result section (L156).

11. 321 – have imposed.

>>> This has been updated.

12. Line 323 – no need to explain Kelch13 interacting candidate… as it is already mentioned in the results.

>>> This has been updated.

13. Line 328ff: Other polymorphisms that appeared to be favored in the Guyana landscape were associated with potential resistance to artemisinin. It seems that this is pure speculation.

>>> In this study, we observed the change of frequency of certain polymorphisms. We believe that the surveillance of this polymorphisms is of interest to the community as pointed by reviewer #2 in Comment #23. As discussed above removing Table 2 from the main text seems appropriate but we followed Comment #23 and added a few references to the polymorphisms uncovered.

14. 333 – should this be the Guiana shield?

>>> True, we made the change. Thank you.

15. Plasmepsin 2 (pfpm2) and pfpm3 – the abbreviation needs to be done when it first appeared. Also see the method part.

Please also check ART (for artemisinin) – abbreviation started in Discussion.
TES: sometimes it is spelled out (e.g., 408), sometimes it is TES (e.g., 409)

>>> This has been corrected. Plasmepsin 2 (pfpm2) now first appears in L107, ART is first introduced in L70 and TES appears in L79 and the abbreviation is kept throughout.

16. 354 – needs to be "single nucleotide polymorphism" as copy number variation is also a polymorphism.

>>> This has been updated.

17. Line 410ff – was the modeling done for a scenario of low endemicity?

>>> No, the modeling was done in high-endemic districts of Rwanda. The approach allowed to prevent the expansion of *pfk13* mutations in the country. This is why this approach of deploying multiple first-line therapies which is similar to antimalarial usage in Guyana could also be interesting to be considered in low endemicity contrasting with the monotherapy approach which led to the emergence of *pfk13* C580Y in SouthEast Asia.

18. Plasmepsin 2/3 CNV was estimated using qPCR. Did WGS identify the CNVs? If the CNV signals were evident from the WGS analysis, what about mdr1 copy number?

>>> It's a reasonable question but due to the uneven coverage resulting from sWGA sequencing, interpreting coverage results for CNV is unreliable, which is why we estimated plasmepsin ⅔ CNV using qPCR. We could not trust solely coverage results and did not explore other CNV for genes like mdr.

Reviewer #2

19. The notation used in the manuscript is sometimes confusing, with clones, clonal components (same as clones?), highly related clusters, and spatial clusters. Perhaps consider calling the spatial clusters something else, so as not to confuse with the clusters based on relatedness? Phrases like clone size were also a bit confusing, since a clone itself would not have a size. I assume clone size refers to abundance of that clone?

>>> We agree about the confusion. We now refer to clonal component simply as 'clone' starting from L126. We then replace all the occurrences of clonal component to clone. We also have changed the term 'spatial clusters' throughout the manuscript to 'epidemiological zones.' We agree regarding the confusion behind clone size and we updated the term to clonal abundance.

20. The spatial analysis and results seem to be underemphasized in the manuscript in general. In addition, some clarification is needed regarding how the spatial analysis was used to infer where an individual acquired their infection. If an individual was diagnosed in one area but indicated travel to another area, how was the source of infection determined? It is very difficult to know the exact location/time of when an individual may have been infected.

>>> Patient travel history records used to infer geographic infection source. Details have been

added to the method section to explain the inference where the patient acquired the infection (L488-494). The diagnoses were inferred from the recollection of patient travel history within the previous two weeks and these inferences are not perfect. This limitation is noted in the discussion (L358-360).

21. It would be helpful for the authors to address whether there might be any "batch" effects that could confound temporal associations, given that the samples from the different time points were processed differently (i.e., samples from one time point underwent sWGA while the others did not). Some information showing similar levels of coverage, particularly in the regions of the assessed drug resistance loci would be helpful in ensuring that the temporal trends aren't a result of technical artefacts.

>>> All samples were collected from dried blood spots and selective whole genome amplification. However, DNA extraction was performed using two approaches but library preparation and sequencing approaches were the same. We acknowledge the potential risk for batch effects within the dataset and we inspected the two datasets. We added a supplementary figure (Fig. S2) with the average coverage for the two temporal datasets and we made a note in the methods (L520-524).

22. In general, the discussion section/interpretation of the results could be expanded and/or better linked to the observed results. For example, the link between the statement in lines 301-303 that "Stochastic processes with intermittent recombination appear to be the dominant mechanism driving clonal diversity rather than a selective advantage obtained from particular polymorphisms favoring a specific clonal background" and the presented data is not completely clear. Although this may be true, it would be helpful for the authors to state explicitly which results/lines of evidence suggest that stochastic processes rather than selective advantage are driving the observed patterns.

>>> Clonal turnover within Guyana appears as a result of stochastic processes, although there is evidence of selection (i.e., reduction of *pfcrt* C350R prevalence or Table S2). The primary evidence for this inference is the observation that resistance mutations are not markedly associated with large clonal persistence and/or abundance. The clonal persistence of the observed *pfK13* C580Y clone is not remarkable, relative to clones bearing mutations of similar frequency (Fig. 4 A & B). We have made this more explicit in the abstract (L25-27) to better convey this message as well as at the beginning of the discussion (L337-341) which now reads: "As suggested by the general lack of impact of resistance mutations on clonal persistence and abundance (Fig. 3), stochastic processes creating the conditions for intermittent recombination appear to be the dominant mechanism driving clonal dynamics, rather than a selective advantage obtained from particular polymorphisms favoring a specific clonal background."

23. Likewise, the discussion in lines 327-330, particularly the statement "Other polymorphisms that appeared to be favored in the Guyana landscape were associated with potential resistance to artemisinin…" should be expanded, as there are several mutated genes (beyond KIC6) in Table 2 that are either the same as or have overlapping function with genes identified as contributors to artemisinin resistance in the GMS. Examples include the FIKKs, CLAGs, and others.

>>> We agree, rather than this simple statement, we added references to some of these genes (notably the importance of FIKK genes and CLAG8 in malaria pathology). This addition conveys the importance of monitoring the rise of the potential polymorphism in the region (L372-375).

24. Also, in the discussion of selection by ACT partner drugs, you might consider contrasting these results with the PfCRT/pfpm2 copy number dynamics observed in the GMS, where copy number decreased rapidly after reduction in DHA-PPQ pressure, but PfCRT mutation prevalence remained high (see Shrestha et al., JID, 2021). This study also showed a "decoupling" of the plasmepsin amplification and the PfCRT mutations associated with piperaquine resistance, consistent with what is observed in this study.

>>> Thank you for the suggestion. We have added a reference to the study in the discussion L391-393.

25. The statement in lines 416-417 is not quite accurate – artemisinin resistance in the GMS started as a soft sweep with multiple K13 mutations emerging on different genetic backgrounds – later C580Y appeared to outcompete the other variants in the eastern GMS, but not the western GMS, where different K13 mutations predominate.

>>> Thank you for the comment. We made the distinction between eastern and western GMS (L471) and corrected this statement throughout the manuscript (L106).

26. Line 128 – the subheading denotes Temporal and Spatial dynamics, but the spatial dynamics are not discussed at all in this section.

>>> The spatial analyses were initially in this section but it was tedious to read without bridging forward important information. Therefore, we moved the spatial analysis to supplementary. We added a link to the supplementary information (L169-170).

27. Were all sequenced isolates from clinical infections (as opposed to asymptomatic infections observed through active surveillance)? If so, it would be good to note this in the methods section.

>>> Indeed, these isolates were collected from clinical infections. This has been updated to make this statement clearer in the methods section (L481-482). It now reads “clinical samples collected from symptomatic individuals seeking treatment and diagnosed with malaria infection”.
