# Supplementary material for: Temporal and spatial dynamics of Plasmodium falciparum clonal lineages in Guyana
Source: PLoS Pathog. 2024 Jun 13;20(6):e1012013. doi: 10.1371/journal.ppat.1012013 (PMC11206942; doi:10.1371/journal.ppat.1012013)
Supplement: Attachment — Submitted filename: Comments.docx [file ppat.1012013.s003.docx]

In this manuscript, Vanhove et al. aimed to determine the population dynamics of P. falciparum in Guyana by sequencing the genomes of a large number of parasite isolates (1727) collected over a 5-year period (2016-2021). They found a clonal population structure of the parasite, comprising 13 highly related clusters. The dynamics of the clones were also followed temporally and spatially. The study also followed the occurrence of drug resistance markers, especially pfk13 C580Y (artemisinin partial resistance), and pfcrt C350R – plasmepsin2/3 amplification (piperaquie resistance). The study presents exciting findings that are suitable for the journal’s audience. While I am not concerned about the study design and data analysis, I have the following comments on the data presentation.

1. Data were not presented sequentially and were hard to follow. For example, in line 194ff, the authors described that C#134 was found in Mid Essenquibo but also found in other spatial clusters (Fig. 3). But Fig 3 illustrated the temporal dynamics of the genetically related clusters. Then, the authors mentioned in line 196ff that C#137 occurred in 9 spatial clusters (Fig. 6) (Same for line 199). But Fig 6 shows selection signals on different chromosomes. The data need to be presented sequentially, not jumping from fig 3 to fig 6. Then, the authors presented data for Fig 7 (line 213ff), and finally touched upon the selection landscape (Fig. 6, line 249ff).
2. Figure 1. Please use a white background to mark the sites, since the color scheme in a dark green background is difficult to view. There are also samples marked in black in Fig 1b – do these samples also represent the ones without traveling histories? Where were the samples collected to document the travel histories?
3. Wihle Figure 2 provides a holistic view of the clusters, it would be more illustrative if the authors could use cluster 1 as an example to make a separate cladogram to show the relationships of the different clonal populations and the singletons within this cluster.
4. Figure 3: The labeling of clusters 1-13 on the left of the figure makes it hard to see which parasites are referred. Could you use a “{“ sign to clearly mark the boundary of each cluster?
5. Figure 4 – hard to view and understand: many colors that differ only slightly and overlap extensively make it difficult to follow. Could the authors present a more concise illustration of the main clusters here? Also, please explain what the density figure shows.
6. Line 175: For those who don’t know the geography of Guyana, it is difficult to figure out where Aranka River in Region 7 is located. Please refer to a closer site that is labeled in figure 1.
7. Line 225: Highly related clusters with more than 2 samples displayed a frequency of pfpm2/3 copy number variation of 37.8% - what does this mean?
8. Mutations in drug resistance genes (237ff): in this section, mutations were found in # samples – also provide the % (as in line 240). For all the mutations, please also mention the amino acids for the positions (for example: 350R, like line 246 – G442H)
9. Selection landscape (line 249ff): The authors provided many details on the genes with increased frequencies in the two study periods. Given that the sampling was not systematic, many samples were clonal, mutations may result from stochastic processes instead of drug selection, and none of them were validated genetically, I think this section should be concise without too much speculation. I think Table 2 should be moved to supplement, as it contains a long list of genes, many of which have no known functions or are not linked to drug resistance.
10. Table 1 needs to be cited in results – when the 13 clusters were analyzed. The first time I see this Table was in the Discussion (line 309)
11. 321 – have imposed
12. Line 323 – no need to explain Kelch13 interacting candidate… as it is already mentioned in the results.
13. Line 328ff: Other polymoprhisms that appeared to be favored in the Guyana landscape were associated with potential resistance to artemisinin. It seems that this is pure speculation.
14. 333 – should this be the Guiana shield?
15. Plasmepsin 2 (pfpm2) and pfpm3 – the abbreviation needs to be done when it first appeared. Also see the method part.

Please also check ART (for artemisinin) – abbreviation started in Discussion.

TES: sometimes it is spelled out (e.g., 408), sometimes it is TES (e.g., 409)

16. 354 – needs to be “single nucleotide polymorphism” as copy number variation is also a polymorphism.
17. Line 410ff – was the modeling done for a scenario of low endemicity?
18. Plasmepsin 2/3 CNV was estimated using qPCR. Did WGS identify the CNVs? If the CNV signals were evident from the WGS analysis, what about mdr1 copy number?
